# The Empty Sylvian Fissure Sign: A Novel Non-contrast CT Finding in Moyamoya Disease

**DOI:** 10.7759/cureus.77385

**Published:** 2025-01-13

**Authors:** Ryotaro Imai, Dai Kamamoto, Hikaru Sasaki, Sadao Suga, Masateru Katayama

**Affiliations:** 1 Department of Neurosurgery, Tokyo Dental College Ichikawa General Hospital, Ichikawa, JPN

**Keywords:** emergency medicine, middle cerebral artery, moyamoya disease, non-contrast computed tomography (ncct), sylvian fissure

## Abstract

Background and objective

In Moyamoya disease, occlusion occurs at the terminus of the bilateral internal carotid artery, resulting in poor visualization of the distal middle cerebral artery (MCA) on imaging. This study aimed to investigate whether the absence of the MCA on non-contrast CT images constitutes a characteristic feature of Moyamoya disease.

Methods

Patients who were documented as having "Moyamoya disease" or "suspected Moyamoya disease" for insurance purposes in medical records were selected. Retrospective analysis of their datasets was conducted. Non-contrast head CT images were reviewed to ascertain the presence or absence of the MCA at four specified locations (right proximal, left proximal, right distal, and left distal) within the Sylvian fissure.

Results

Of the 151 patients identified, 23 cases of Moyamoya disease and 35 cases without Moyamoya disease were investigated. The Moyamoya disease cohort exhibited a significantly higher number of absent MCA observations within the four locations (2.65 vs. 0.83, p<0.001). With a threshold of ≥3 locations devoid of the MCA, the sensitivity and specificity of this finding for Moyamoya disease detection were 61% and 94%, respectively.

Conclusions

This study represents the first exploration of non-contrast head CT findings indicative of Moyamoya disease. Although cerebral angiography or MRI is indispensable for definitive diagnosis, a subset of patients may not undergo these detailed examinations. Focusing on the "empty Sylvian fissure sign" on non-contrast head CT images, frequently encountered in acute medical settings, may help detect patients with Moyamoya disease and support further evaluation.

## Introduction

Moyamoya disease, a cerebrovascular disorder of uncertain etiology, is particularly prevalent among the Japanese population and is characterized by progressive occlusion of the circle of Willis and proliferation of anomalous vascular networks known as Moyamoya vessels [1]. While cerebral angiography or MRI is essential for a definitive diagnosis, a subset of cases, including asymptomatic lesions, cannot be diagnosed in the absence of these detailed examinations. In Moyamoya disease, the terminus of the bilateral internal carotid artery is occluded, and the distal middle cerebral artery (MCA) is poorly visualized on imaging. The MCA has a visible vascular structure even on non-contrast CT images, as represented by the hyperdense MCA sign in acute cerebral infarction [2,3]. This study aims to ascertain the extent to which the MCA remains indistinct on non-contrast head CT images in patients with Moyamoya disease.

## Materials and methods

We enrolled individuals who were documented in our institutional electronic medical record from January 2017 to August 2023 to have "Moyamoya disease" or "suspected Moyamoya disease" for insurance purposes. Patients lacking CT images were excluded. Subsequently, a comprehensive investigation was conducted on the patient profiles, diagnostic information, and non-contrast head CT images. Specifically, we verified whether the diagnosis was Moyamoya disease and examined the presence of the MCA across four segments within the Sylvian fissure (right proximal, left proximal, right distal, and left distal) as delineated on non-contrast cerebral CT images. Patients with atherosclerotic lesions or indeterminate diagnoses arising from poor imaging findings were excluded. Furthermore, individuals with Moyamoya disease presenting solely with post-surgical revascularization images and those with unilateral Moyamoya disease were also excluded from the investigation.

The proximal segment of the Sylvian fissure was defined as the linear subarachnoid space visualized on axial CT images parallel to the orbitomeatal line, where the sphenoidal segment of the MCA (M1) courses laterally. Anatomically, the proximal segment corresponds to the sphenoidal compartment of the Sylvian fissure [4], situated between the orbital gyrus and the anterior surface of the planum polare and parahippocampal gyrus [5]. Similarly, the distal segment was characterized as a crescent-shaped subarachnoid space evident on axial CT images, enclosed by the insula, frontal lobe, and temporal lobe, through which the insular segment of the MCA (M2) runs. Anatomically, the distal segment corresponds to the anterior operculoinsular compartment of the Sylvian fissure [4], positioned between the orbital gyrus and the middle and posterior surfaces of the planum polare [5].

We defined the "empty Sylvian fissure sign (ESF sign)" as the absence of MCA vasculature within each segment. Additionally, to quantify the ESF sign, we introduced the "ESF score" signifying the number of Sylvian fissure segments with the ESF sign, which spans from a minimum of zero to a maximum of four. To evaluate the disparity in ESF sign prevalence between the Moyamoya and non-Moyamoya disease cohorts, a Student's t-test was employed for the ESF score. The analysis was conducted using the statistical software Statcel 3 (OMS Publishing, Tokyo, Japan) and independently repeated twice.

## Results

**Figure 1:**
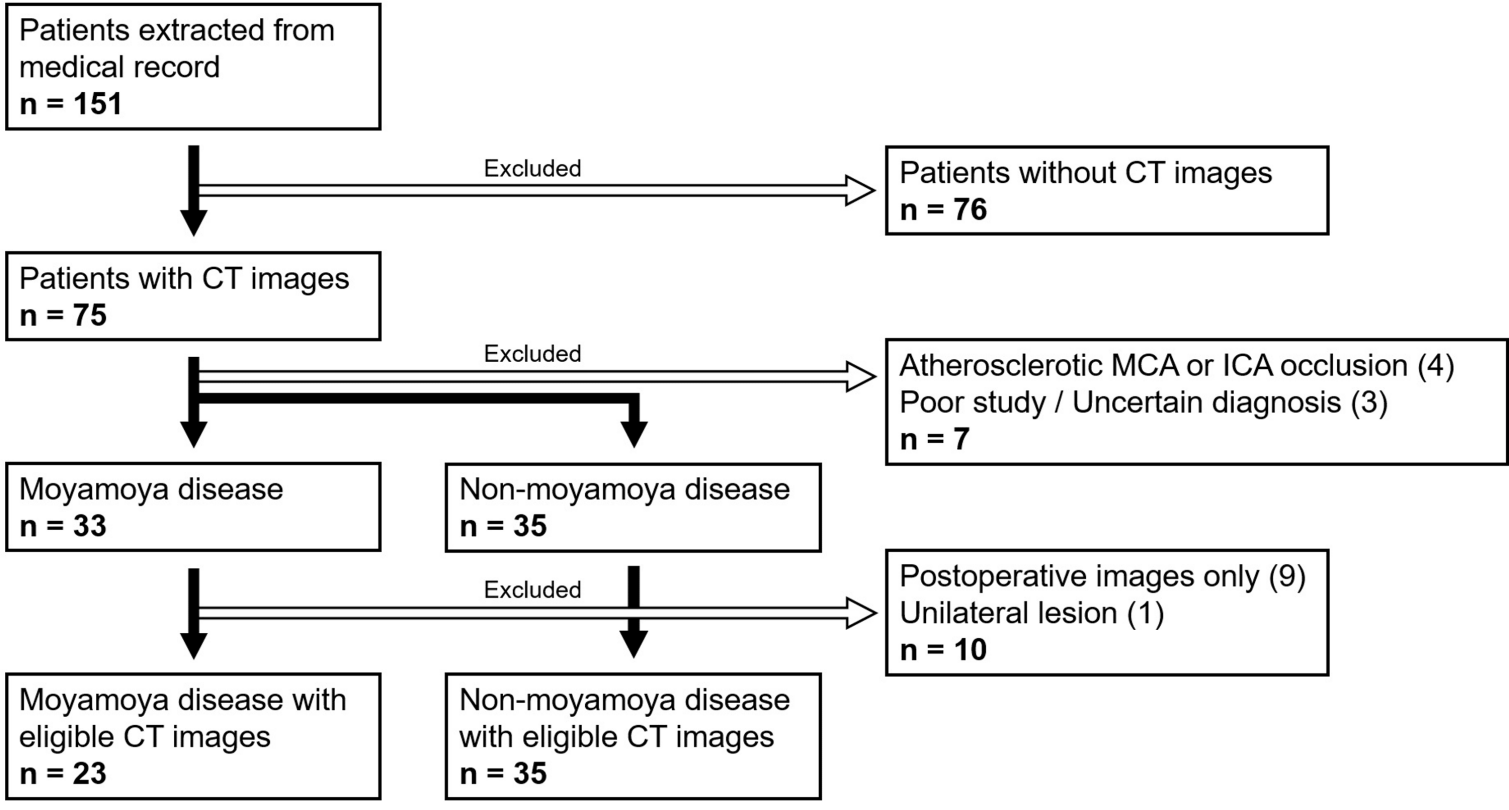
Patient backgrounds Of the 151 enrolled patients, 75 had accessible CT data. Among them, 33 were diagnosed with Moyamoya disease, while 35 were classified as non-Moyamoya cases based on supplementary clinical information. The remaining seven cases consisted of four instances of atherosclerotic occlusion of the MCA or ICA and three cases with indeterminate diagnoses. After excluding patients presenting solely with postoperative images or unilateral lesions, the final Moyamoya and non-Moyamoya disease cohorts comprised 23 and 35 patients with eligible CT images, respectively CT: computed tomography; ICA: internal carotid artery; MCA: middle cerebral artery

Of the 151 enrolled patients, 75 had CT scan data available: 33 were diagnosed with Moyamoya disease and 35 were categorized as the non-Moyamoya disease cohort based on additional clinical findings. The remaining seven cases comprised instances of atherosclerotic occlusion of the MCA or internal carotid artery (four cases) and indeterminate diagnoses (three cases). After excluding patients with only postoperative images or unilateral lesions, the Moyamoya and non-Moyamoya cohorts included 23 and 35 patients with eligible CT images, respectively. Patient backgrounds are summarized in Figure 1.

**Figure 2:**
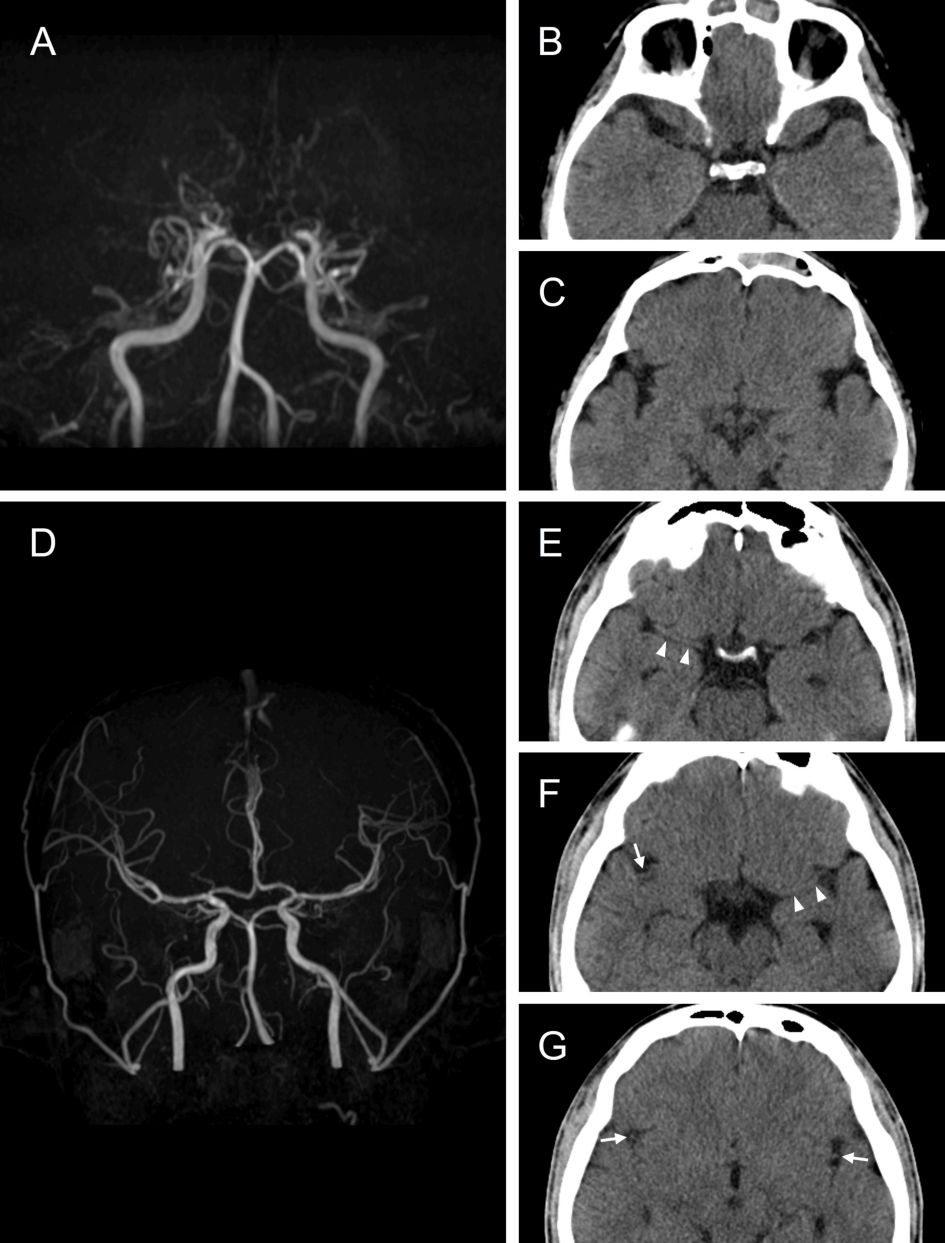
Representative cases with and without the ESF sign (A–C) An illustrative case of a 36-year-old male diagnosed with Moyamoya disease. Magnetic resonance angiography revealed bilateral disappearance of the MCA and proliferation of moyamoya vessels (A). On non-contrast CT images, the absence of MCA vasculature (ESF sign) was noted bilaterally in both proximal (B) and distal (C) Sylvian fissures. The ESF score was calculated to be 4. (D–G) A 20-year-old male without Moyamoya disease. Magnetic resonance angiography demonstrated a normal vascular anatomy (D). Non-contrast CT images depicted discernible MCA vasculature in both proximal (E and F; indicated by arrowheads) and distal (F and G; indicated by arrows) Sylvian fissures. No ESF sign was evident in this case; therefore, the ESF score was calculated to be 0 CT: computed tomography; ESF sign: empty Sylvian fissure sign; MCA: middle cerebral artery

An illustrative case with an ESF sign is shown in Figures 2A-2C. It involves a 36-year-old male diagnosed with Moyamoya disease who exhibited an absence of discernible vascular structures within each segment of the Sylvian fissure, indicative of a positive ESF sign across all four segments, leading to an ESF score of 4. Details of the non-Moyamoya disease group are illustrated in Figures 2D-2G. The representative patient was a 20-year-old male without Moyamoya disease. Here, the MCA vasculature was observable throughout all segments of the Sylvian fissure, resulting in an ESF score of 0. Despite the typical nature of the aforementioned cases, instances of Moyamoya disease with Sylvian fissure segments lacking the ESF sign, and cases of non-Moyamoya disease displaying ESF sign positivity in certain segments were encountered.

Within the cohort diagnosed with Moyamoya disease, the presence of the ESF sign was confirmed in 22 of the 23 cases across some of the Sylvian fissure segments. Conversely, in the non-Moyamoya disease cohort, signs were detected in 19 of the 35 cases. The ESF score, denoting the number of Sylvian fissure segments manifesting the ESF sign, exhibited a statistically significant elevation in the Moyamoya disease group compared to the non-Moyamoya disease group (2.65 vs. 0.83, p<0.001, Table 1). Employing an ESF score threshold of three or greater, the sensitivity and specificity of the ESF sign for detecting Moyamoya disease were determined to be 61% and 94%, respectively. The mean age of the patients showed a significant disparity between the Moyamoya and non-Moyamoya disease cohorts (46.1 vs. 29.6, p=0.004).

**Table 1:** ESF sign-related observations ESF score: empty Sylvian fissure score, i.e., the number of Sylvian fissure segments exhibiting the empty Sylvian fissure sign (ranging from a minimum of 0 to a maximum of 4) ESF sign: empty Sylvian fissure sign

Patient group	Number of ESF signs at each segment				ESF score	P-value
	Proximal Sylvian fissure		Distal Sylvian fissure			
	Right	Left	Right	Left		
Moyamoya disease (n=23)	16	9	20	16	2.65 ± 0.26	<0.001
Non-Moyamoya disease (n=35)	10	3	12	4	0.83 ± 0.17	

## Discussion

Several reports have revealed the imaging findings of Moyamoya disease employing modalities other than CT [6], as well as analyses of hemorrhage and ischemia findings utilizing CT [7]. However, to the best of our knowledge, no plain head CT findings specific to Moyamoya disease have been reported so far. An MRI study indicated that the outer diameter of the MCA is reduced in patients with Moyamoya disease, in contrast to those with atherosclerotic MCA stenosis or occlusion [8]. This finding is consistent with the observation of ESF signs on non-contrast CT images in Moyamoya disease.

CT is commonly used in emergency medicine in Japan, with head CT constituting the majority of these procedures [9,10]. However, the diagnostic significance of head CT for conditions such as syncope remains uncertain [11,12]. Symptoms such as headache, seizures, and altered consciousness are frequently manifested in patients with Moyamoya disease (34%, 4%, and 21%, respectively) with syncope presentations being common [13-15]. In cases where patients exhibit these symptoms but non-contrast head CT scans reveal no significant abnormalities, Moyamoya disease may go undetected without further assessment via contrast-enhanced CT or MRI. Identification of the absent MCA vasculature in the Sylvian fissure (termed the ESF sign) on non-contrast CT images may help detect patients with Moyamoya disease, prompting thorough examination and appropriate follow-up. According to our current analysis, an ESF score of 3 or higher may suggest the likelihood of Moyamoya disease, necessitating further investigation.

In the majority of cases, the documented disease name "suspected Moyamoya disease" served as the insurance-related disease name for conducting MRI in response to chief complaints such as headache, dizziness, etc. Notably, the non-Moyamoya disease group comprised individuals who were no longer suspected of having Moyamoya disease and indeed constituted a control cohort devoid of any association with Moyamoya disease.

In the present analysis, the mean age of patients was significantly higher in the Moyamoya disease group. However, diverse conjectures may have arisen based on this observation. Hemoglobin levels decline with advancing age [16], and there is a positive correlation between hemoglobin levels and CT values [17,18]. Thus, it is possible that the CT values of the MCA were diminished in the Moyamoya disease group, rendering MCA visualization challenging on non-contrast CT images. Conversely, because the Sylvian fissure enlarges with advancing age [19], this expansion theoretically facilitates the identification of vascular structures within the fissure in elderly patients. The predominance of ESF signs in the Moyamoya disease group, which had a higher mean age, emphasizes the robustness of this observation.

Limitations

This study has a few limitations. The present investigation was conducted without blinding; therefore, the researchers reviewed the CT images while being aware of the Moyamoya disease diagnostic status. Failure to mitigate observer bias was the primary constraint of this study. Another limitation related to the inclusion of images sourced from other hospitals or acquired using different equipment, which resulted in non-standardized imaging protocols. Moreover, in this study, we could not assess the correlation between ESF signs and the Suzuki grading system, since not all patients underwent cerebral angiography and it was not feasible to gather adequate information on Moyamoya disease grade from the records. Further research is warranted to gain deeper insights into the topic.

## Conclusions

The ESF sign, characterized by the absence of the MCA in specified locations on head non-contrast CT images, demonstrates significant potential as a diagnostic marker. Despite the necessity of advanced examinations such as cerebral angiography and MRI for definitive diagnosis, the ESF sign may serve as a valuable preliminary tool in detecting Moyamoya disease. This approach in emergency medicine could ensure timely evaluation for patients who might otherwise not undergo further investigations.
